# Impact of Laboratory Test Use Strategies in a Turkish Hospital

**DOI:** 10.1371/journal.pone.0153693

**Published:** 2016-04-14

**Authors:** Fatma Meriç Yılmaz, Rabia Kahveci, Altan Aksoy, Emine Özer Kucuk, Tezcan Akın, Joseph Lazar Mathew, Catherine Meads, Nurullah Zengin

**Affiliations:** 1 Yildirim Bayezid University, Department of Biochemistry, Ankara, Turkey; 2 Ankara Numune Training and Research Hospital, Health Technology Assessment Unit, Ankara, Turkey; 3 Ankara Numune Training and Research Hospital, Department of Microbiology, Ankara, Turkey; 4 Ankara Numune Training and Research Hospital, Department of General Surgery, Ankara, Turkey; 5 Advanced Pediatrics Centre PostGraduate Institute of Medical Education and Research, Chandigarh, India; 6 Brunel University, Health Economics Research Group, Uxbridge, United Kingdom; 7 Ankara Numune Training and Research Hospital, Department of Oncology, Ankara, Turkey; University of Florence, ITALY

## Abstract

**Objectives:**

Eliminating unnecessary laboratory tests is a good way to reduce costs while maintain patient safety. The aim of this study was to define and process strategies to rationalize laboratory use in Ankara Numune Training and Research Hospital (ANH) and calculate potential savings in costs.

**Methods:**

A collaborative plan was defined by hospital managers; joint meetings with ANHTA and laboratory professors were set; the joint committee invited relevant staff for input, and a laboratory efficiency committee was created. Literature was reviewed systematically to identify strategies used to improve laboratory efficiency. Strategies that would be applicable in local settings were identified for implementation, processed, and the impact on clinical use and costs assessed for 12 months.

**Results:**

Laboratory use in ANH differed enormously among clinics. Major use was identified in internal medicine. The mean number of tests per patient was 15.8. Unnecessary testing for chloride, folic acid, free prostate specific antigen, hepatitis and HIV testing were observed. Test panel use was pinpointed as the main cause of overuse of the laboratory and the Hospital Information System test ordering page was reorganized. A significant decrease (between 12.6–85.0%) was observed for the tests that were taken to an alternative page on the computer screen. The one year study saving was equivalent to 371,183 US dollars.

**Conclusion:**

Hospital-based committees including laboratory professionals and clinicians can define hospital based problems and led to a standardized approach to test use that can help clinicians reduce laboratory costs through appropriate use of laboratory tests.

## Introduction

Reducing healthcare costs, with the maintainance of patient safety and improved quality, is one of the main targets in most healthcare reform efforts. It is often difficult to decide how to achieve this goal. Eliminating unnecessary laboratory tests and procedures is one good place to start. It has been reported that approximately $6.8 billion of medical care in the United States has involved unnecessary testing and procedures that do not improve care and may even harm the patient [1]. The American Society for Clinical Pathology dedicated their April 2012 edition of Critical Values to the issue of appropriate laboratory testing, and pointed out the aim as ‘‘right test, right patient, right time, at the right cost” [2].

It is important to prioritise hospital-based strategies to reduce healthcare costs. ANHTA is the first hospital based Health Technology Assessment (HTA) Unit in Turkey. The unit aims to support hospital managers in evidence-based investment or disinvestment decisions regarding health technology use in the hospital. Hospital based committees, including laboratory professionals and clinicians, can define hospital based problems, which can lead to standardized approaches. Hospital based HTA units have a crucial role in working with the related professionals to facilitate an evidence-based approach in the process, which would further lead to improved quality with reduced costs.

In this study, our aim was to define and process strategies to rationalize laboratory use in Ankara Numune Training and Research Hospital (ANH) and calculate impact and potential savings in health-care costs.

## Methods

A collaborative action plan was defined by the hospital managers. Joint meetings with ANHTA and laboratory chairs were set; the joint committee invited relevant staff for input, and hospital laboratory efficiency committee was created, including clinicians from internal medicine, general surgery, family medicine, emergency department and the laboratory directors of biochemistry and microbiology departments. Literature was reviewed in order to identify strategies used to improve laboratory efficiency. Strategies that would be applicable in local setting were identified for implementation, processed and impact on clinical use and costs was assessed for 12 months.

Implementations were decided and processed as detailed below:

### Taking a picture about the current status and increasing the awareness

1. A hospital meeting was conducted in order to create an awareness and the doctors from various departments were informed about the appropriate use of laboratory tests. The examples of the overused tests were shared and discussed during the meetings. The impact of the laboratory costs, the potential harms of ordering an unnecessary test for a patient, the importance of increasing the clinician-laboratory interaction was emphasized.
2. A follow up procedure was planned and a laboratory use report, including mean number of ordered tests per patient and total laboratory cost of every department was prepared. Laboratory use reports were sent to the departments every month.
3. A review was performed to understand how various laboratory tests were used and whether the use was appropriate according to the guidelines or evidence provided in the literature.

### Literature search about evidence based laboratory practices and identification of strategies that would be applicable in local setting

4 Literature was reviewed in order to identify strategies used to improve laboratory efficiency and the implementations reported in the literature were listed. Although a formal systematic review was not undertaken as this was outside the scope of this study, our primary objective was to identify any relevant information related to the objective of our study. We looked for systematic reviews and health technology assessment reports as well as any trials that would provide additional information about techniques to improve laboratory use. We searched PubMed and the Cochrane Library, using “(laboratory test) AND (misuse OR overuse)” as search terms. The databases of health technology assessment agencies were manually searched for relevant references. We also searched for relevant citations from the articles we initially selected, to locate any additional studies of interest. The inclusion criteria were systematic reviews/reviews/randomized controlled trials/HTA reports (Study Design) of interventions/strategies (Intervention) to reduce the misuse or abuse of laboratory tests (Outcome) ordered by physicians in hospital settings (Population/Problem). Two researchers reviewed all relevant papers to identify different techniques and interventions to affect laboratory use and summarized their findings.
5 After the review of techniques, the barriers which can block the process during implementation and possible solutions were discussed by the committee.

### Monitoring of the effects and evaluation of the economic impact

6 The number of ordered tests and total laboratory costs of the hospital were followed for one year by monitoring the laboratory test amount, between March 2013-March 2014. Numbers of the ordered tests were compared with the previous year numbers for every month. The amount of this decrease was calculated as the Reduction% (R% = [(Sum of the tests between March 2012-March 2013)- (Sum of the tests in March 2013-March 2014)/(Sum of the tests between March 2012-March 2013)]*100). The total impact was calculated at the end of the one-year-follow up period.

## Results

This study was run in ANH, which is in service in Ankara, Turkey since 1881. ANH is a reference hospital with great experience in research and training. It has 1200 bed capacity and service is provided by over 5000 staff. Health care services run in 38 different specialties; and residency trainings are given in 31 specialties. It had 218.866.322 TL budget in 2013 and 235.398.498 in 2014 (over 80 million euros per year in average). Outpatient admissions were 962.589 patients in 2013 and 1.013.300 patients in 2014. Highest number of admissions were to internal medicine and emergency departments. Overall mortality rate in the hospital during the year preceding the intervention was 0.028 and was unchanged the following year. Similarly, the mean (SD) length of hospitalization was 6.2 (0.3) days during the first year and 6.3 (0.2) during the second year of the study.

### Search Results

**Fig 1:**
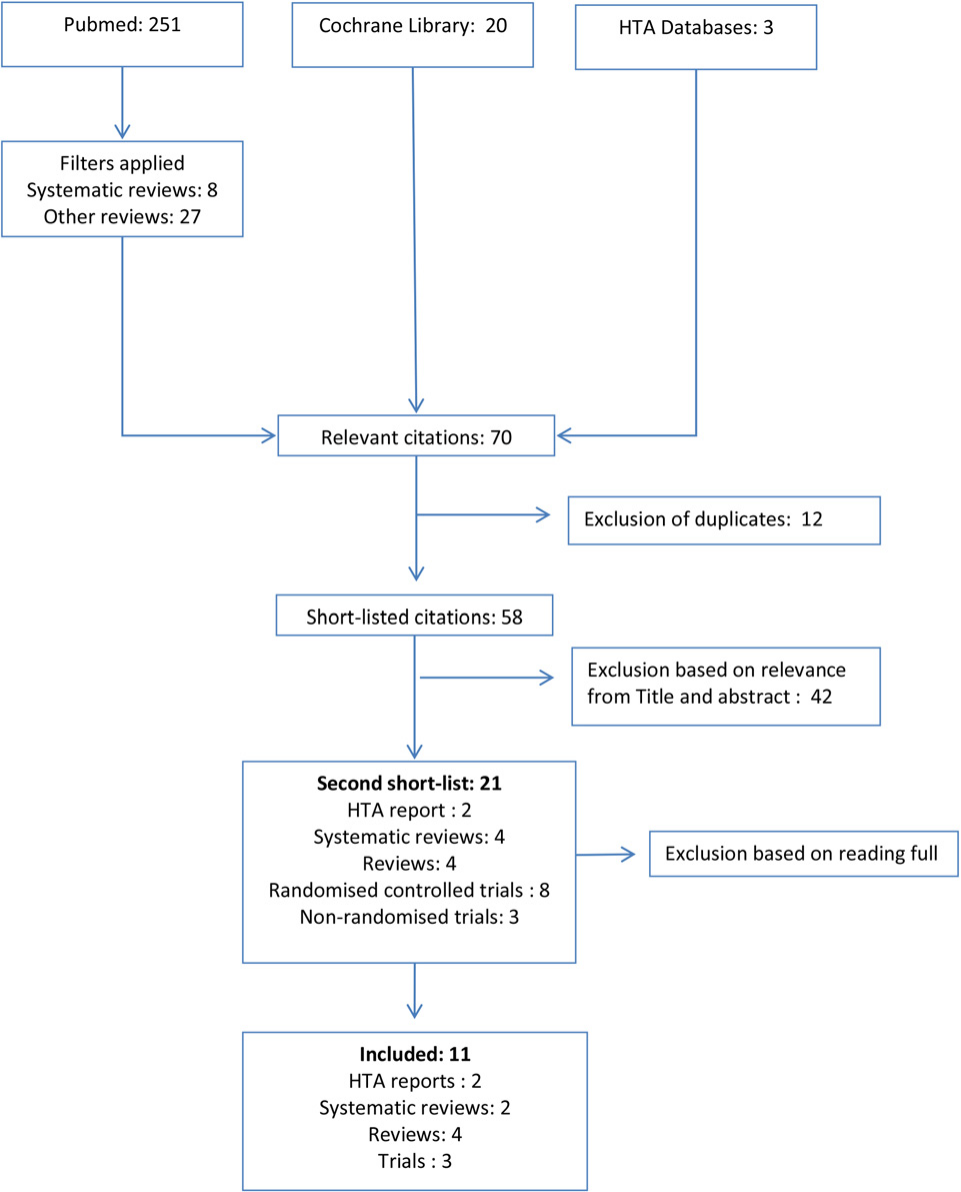
Flow diagram showing literature search.

The literature review through PubMed identified 251 potentially relevant articles. A filter for systematic reviews revealed 8 and a filter for reviews revealed 27 hits. 12 randomized controlled trials were also identified. A review of the Cochrane Library revealed 20 trials. No HTA report was identified through this search. The manual search of databases of HTA agencies revealed three related reports. There were 12 overlaps, which left us 58 papers. 42 papers were excluded through a first overview of titles, ending up with 2 HTA reports, 4 systematic reviews, 4 reviews, 8 randomised controlled trials and 3 non-randomised trials. Exclusion of non-relevant papers based on full reading of the papers finally left us 2 HTA reports, 2 systematic reviews, 4 reviews and 3 trials. Flow diagram and list of the included studies are given in Fig 1 and Table 1.

**Table 1:** List of the included studies.

***HTA Reports***
Canadian Agency for Drugs and Technologies in Health Rapid Response Report:. Techniques to improve the use of diagnostic laboratory test ordering: Clinical Evidence. 12 March 2013.
Canadian Agency for Drugs and Technologies in Health. Health Technology Inquiry Service HTA Report: Diagnostic Test Ordering: Techniques to improve use. 13 May 2010.
***Systematic Reviews***
Hauser RG, Shirts BH. Do we now know what inappropriate laboratory utilization is? An expanded systematic review of laboratory clinical audits. Am J Clin Pathol. 2014; 141(6):774–83.
van Walraven C, Naylor CD. Do we know what inappropriate laboratory utilization is? A systematic review of laboratory clinical audits. JAMA. 1998;280(6):550–8.
***Reviews***
Axt-Adam P, van der Wouden JC, van der Does E. Influencing behavior of physicians ordering laboratory tests: a literature study. Med Care. 1993;31(9):784–94.
Smetana GW, Macpherson DS. The case against routine preoperative laboratory testing. Med Clin North Am. 2003 Jan;87(1):7–40.
Levick DL, Stern G, Meyerhoefer CD, Levick A, Pucklavage D. "Reducing unnecessary testing in a CPOE system through implementation of a targeted CDS intervention". BMC Med Inform DecisMak. 2013 Apr 8;13:43
Ferraro MJ. Effect of diagnosis-related groups on diagnostic methodology in the hospital laboratory. DiagnMicrobiol Infect Dis. 1986;4:135S-142S.
***Trials***
Calderon-Margalit R, Mor-Yosef S, Mayer M, Adler B, Shapira SC. An administrative intervention to improve the utilization of laboratory tests within a university hospital. Int J Qual Health Care. 2005 Jun;17(3):243–8.
Wong ET, McCarron MM, Shaw ST Jr. Ordering of laboratory tests in a teaching hospital. Can it be improved? JAMA. 1983 Jun 10;249(22):3076–80.
Isouard G. A quality management intervention to improve clinical laboratory use in acute myocardial infarction. Med J Aust. 1999 Jan 4;170(1):11–4

The findings were later discussed by the committee to identify which techniques would be implemented in the local setting.

#### Techniques use review results

The CADTH reports [3,4] highlighted the utilization of computerized decision support systems observing that use of such technology improved physician prescribing behavior. One trial comparing watchful waiting rather than direct ordering of tests concluded that this was a practical approach to optimize laboratory use. Audit feedback was also presented as an appropriate strategy for limiting the use of laboratory tests. The INESSS report [5] suggested the use of Information technology (IT) for decision support algorithms, development of guidelines, modification of prescriber behaviors, and appropriate legislation. Similar use of IT was suggested by Levick et al, whereby computerized alerts (red flags) could warn the clinicians and the laboratory specialists when certain tests were ordered excessively [6]. Ferraro recommended close collaboration between the physicians ordering tests and the laboratory personnel providing the services [7]. Organizational strategies including a focus on Total Quality Management have also been presented as a method to optimize use of laboratory services. In one study, the investigators highlighted the role of administrative modifications such as limiting the availability of certain laboratory services based on their frequency, education of hospital staff, and feedback to streamline the process [8].

#### Test use review results

All of the laboratory orders for a three month period were investigated in order to understand the tendencies of the clinicians which might be quite different for every hospital. Overused (ordered more commonly than expected e.g folic acid, LDH, chloride orders which have little clinical impact on clinical decision except specific patients) or misused tests (e.g. HBeAg test order for a HBsAb positive patient, TORCH (toxoplasmosis, rubella, cytomegalovirus, herpes) IgG and IgM testing for every pregnant women, preoperative hepatitis and HIV testing, free PSA testing independent from total PSA levels) were reviewed. During this review, Na-K-Chloride tests were ordered together in %98.5 of the patients, GGT-ALP-LDH tests were ordered together in 86% of the patients, total PSA and free PSA were ordered at the same time in 98% of the patients. The results of the test use review at ANH showed that major use was in internal medicine. The mean number of tests per patient was 15.8. Laboratory use differed enormously among clinics (5–28 tests per patient). The budget impact of all tests per year was 4 433 902 $. Overuse of chloride, lactate dehydrogenase, free PSA (prostate specific antigen), folic acid, hepatitis and HIV tests, and considerable variation from guideline recommendations for preoperative routine testing were observed. For example chloride was observed to be ordered as a part of Na-K-Cl electrolyte panel and was ordered for every patient who had an electrolyte testing. Free PSA was ordered independently from the total PSA level although it is known to be valuable for only the patients whose total PSA levels are within the gray zone (2–10 ng/mL). Folic acid was ordered for the majority of patients who had been tested for Vitamin B12 levels. The reason for the overuse of these tests was thought as the tendency of the clinicians to order specific tests attached to another (e.g. ordering chloride with sodium and potassium or folic acid with Vitamin B12). Clinicians were observed to use test panels which they constitute for quick pick up of the laboratory tests and these panels have thought to be a major cause of unnecessary testing for ANH.

### Strategies decided to be implemented based on consensus of the committee members

1. Education of the clinicians about evidence based practices [9,10]. This strategy was implemented through hospital internal meetings.
2. Informing clinicians about the laboratory costs [11,12]. A laboratory use report including mean number of ordered tests per patient and total laboratory cost of every department was sent to each department every month.
3. The rearrangement of the computerized test ordering page [13]. This rearrangement was performed for the tests which were detected as overused tests during the use review. Test ordering page was divided into two separate pages which were in different screens on the computer and the tests which give less information, ordered more than expected or observed to be mis-used were taken to the second page.
4. Using test panels during the test order was determined as a major problem causing overuse. Therefore, the use of the test panels was forbidden, after informing the clinicians.
5. The integration of prompting notes to the computerized test ordering page [14]. We implemented a prompting note to give information when the same test had been ordered within the previous week.

### Monitoring and impact evaluation

We started to implement the defined strategies by February 2013. The first strategy of the above was implemented in February while the rest of the strategies were implemented by March 2013. We did not follow the the impact of the strategies separately in order not to extend the following time. We aimed to obtain a maximum impact with the combination of the five strategies. The impact on laboratory use and costs were monitored with the follow up of the test numbers and impact was evaluated for the following 12 months after March 2013. There was a significant decrease in the number of the tests after the decided interventions.

All of the panels involving electrolyte testing included Na-K-Chloride tests independent from the diagnosis, although chloride test is recommended to be used in specific conditions [15]. After prohibition of the panels, the chloride test was moved to the second test ordering page, which inhibited the quick-pick of the test and a reduction of 51.3% was observed. The same strategy provided a 44.2% reduction for free PSA and 67.7% reduction for folic acid. The reason for overuse of these tests just seemed to be an ordering habit without a significant scientific reason, and separation of Cl from Na-K, folic acid from vitamin B12 and free PSA from total PSA was thought to decrease this kind of use.

A significant overuse in ordering of TORCH (IgG antibodies measured by ELISA against toxoplasmosis, rubella, cytomegalovirus, herpes) testing has been observed during the use review. Moving these tests to the second page resulted in reduction of 78.9%, 85.0% and 79.8%, respectively for IgG antibodies to cytomegalovirus, toxoplasmosis and rubella. The reason for the overuse of ELISA tests was thought to be related to insufficient medical knowledge to order and interpret the ELISA tests and further training of residents has been planned.

**Fig 2:**
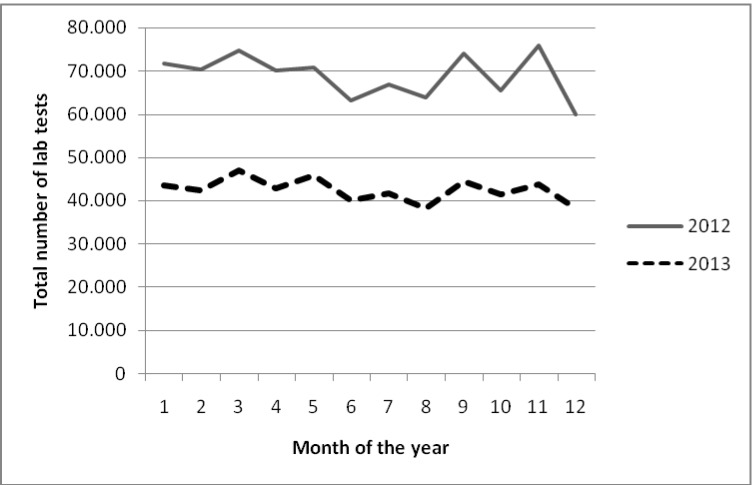
Total number of laboratory tests moved to the second page per month.

The total test numbers of the parameters which were moved to the second page in comparison with the previous year’s numbers are given in Table 2. Use of all of the tests which were moved to the second page decreased significantly (between 12.6–85.0%). The comparison of the sum of these tests with the previous year is demonstrated in Fig 2. The most significant decrease was observed in toxoplasmosis IgG at 85,0%. The one year impact of the study was calculated as a saving of $371 183.

**Table 2:** Numbers of tests moved to the second page in the computerized test ordering page during the follow up period and comparison with the previous year’s numbers. (%R = Percentage of reduction in test numbers).

		March	April	May	June	July	August	September	October	November	December	January	February	%R
**Number of Patients**	**Before**	85.808	81.252	84.307	78.261	76.227	68.143	75.434	72.065	83.167	81.791	88.283	87.851	
	**After**	90.621	89.720	91.241	80.448	80.238	72.325	81.994	71.821	87.059	90.317	90.412	87.104	
**Uric Acid**	**Before**	12.186	11.460	12.121	11.798	12.157	9.438	10.568	10.240	12.599	11.372	13.375	9.573	**42,5**
** **	**After**	6.293	5.670	6.931	6.268	7.052	5.664	6.401	5.432	6899	7.412	7.820	6.921	** **
**Lipase**	**Before**	706	720	752	715	680	699	645	605	842	593	595	563	**75,2**
** **	**After**	179	142	138	138	129	151	145	155	221	211	229	171	** **
**Chloride**	**Before**	19.806	19.818	21.015	19.668	20.376	19.225	19.701	19.367	20.494	14.861	20.177	16.502	**51,3**
	**After**	9.491	8.794	10.017	9.484	10.144	8.903	7.988	8.783	8391	10.127	10.678	9.608	
**LDH**	**Before**	15.444	16.257	17.378	16.411	16.789	15.361	15.252	15.343	16.159	11.745	16.672	13.662	**29,5**
** **	**After**	10.470	9.819	10.912	10.786	11.272	11.033	10.393	10.295	11122	12.322	12.641	10.432	** **
**Pre Albumin**	**Before**	499	470	394	448	458	406	425	394	495	506	2.079	723	**78,5**
** **	**After**	251	147	176	121	112	123	112	101	116	84	135	94	** **
**ASO**	**Before**	1.431	1.366	1.510	1.335	1.207	973	1.284	1.155	1.528	1.007	1.092	1.012	**48,6**
** **	**After**	635	642	828	634	667	555	745	502	710	586	602	558	** **
**RF**	**Before**	3.258	2.923	3.342	3.128	2.904	2.225	2.593	2.556	3.282	2.208	2.700	3.174	**27,5**
** **	**After**	2.497	2.271	2.518	1.821	1.790	1.714	1.864	1.450	2385	2.058	2.316	2.167	** **
**Ig G**	**Before**	609	631	706	604	596	485	556	447	649	410	553	534	**27,8**
** **	**After**	382	375	390	359	441	411	467	340	473	468	380	408	** **
**Ig M**	**Before**	596	628	703	608	600	483	555	436	642	400	551	526	**29,0**
** **	**After**	378	373	381	359	426	388	457	336	468	451	374	386	** **
**Ig A**	**Before**	564	599	645	580	557	472	535	418	639	378	547	524	**23,1**
** **	**After**	383	377	417	366	446	410	471	348	482	471	395	403	** **
**C3**	**Before**	465	430	433	444	397	404	398	318	471	305	427	378	**12,6**
	**After**	309	332	431	363	382	390	360	233	392	350	456	259	** **
**C4**	**Before**	478	445	440	460	406	413	420	339	485	325	449	388	**19,7**
** **	**After**	313	330	436	368	371	288	311	224	378	340	443	254	** **
**Free PSA**	**Before**	1.277	1.226	1.274	1.022	932	782	945	957	1.216	954	1.533	1.241	**44,2**
** **	**After**	911	632	600	524	514	455	501	448	714	654	794	704	** **
**CA 15–3**	**Before**	1.401	1.354	1.405	1.228	1.209	990	1.276	1.081	1.306	1.065	1.573	1.082	**29,7**
** **	**After**	955	917	911	801	892	741	913	668	1020	946	939	819	** **
**CA 125**	**Before**	1.320	1.325	1.340	1.137	1.099	928	1.194	967	1.262	960	1.593	1.037	**33,8**
** **	**After**	858	818	805	698	839	590	780	532	943	833	822	863	** **
**CA 19–9**	**Before**	1.748	1.612	1.613	1.376	1.359	1.172	1.496	1.211	1.624	1.239	1.791	1.386	**26,9**
** **	**After**	1.166	1.113	1.081	969	1.126	805	1.152	846	1236	1.113	1.142	1.128	** **
**Folic Acid**	**Before**	5.756	5.283	5.688	5.406	5.467	4.901	4.906	4.659	5.944	3.659	6.459	3.779	**67,7**
** **	**After**	1.472	1.462	1.825	1.678	1.950	1.624	2.055	1.229	1707	1.784	1.781	1.445	** **
**Anti TG Ab**	**Before**	884	802	780	693	623	729	650	547	836	600	850	669	**22,9**
** **	**After**	437	496	561	526	870	612	651	368	516	540	510	588	** **
**Anti HBs**	**Before**	2.274	2.107	2.224	2.262	2.170	2.053	2.510	1.986	2.608	1.827	1.992	2.201	**62,3**
** **	**After**	879	881	964	690	750	670	759	746	895	892	977	772	** **
**CMV IgG**	**Before**	234	207	213	207	196	277	198	175	232	173	225	208	**78,9**
** **	**After**	53	54	41	40	40	37	37	34	46	52	55	48	** **
**Toxo IgG**	**Before**	342	321	326	318	327	374	331	312	393	282	310	330	**85,0**
** **	**After**	70	58	43	39	44	44	44	44	43	65	53	49	** **
**Rubella IgG**	**Before**	330	220	320	307	313	371	321	299	384	278	306	327	**79,8**
** **	**After**	70	59	43	50	46	60	60	46	72	90	89	77	** **
**TOTAL**	**Before**	71.608	70.204	74.622	70.155	70.822	63.161	66.759	63.812	74.090	65.461	75.849	59.819	**38,5**
	**After**	43.426	42.200	46.915	42.707	45.820	39.996	41.525	38.208	44.266	41.272	43.631	38.154	** **

## Discussion

This study reflects the first results from an ongoing HTA program on appropriate test use in ANH. Our one year experience showed that a collaborative plan might have significant effects in reducing laboratory costs. Although we neither documented and quantified misuse nor formally used benchmarks, our baseline data provided clear signals of overuse of basic laboratory tests based on the literature search, also supported by variation across hospital units, giving support to our cost-containment action.

More than half of the clinical decisions have known to be influenced by laboratory results [16]. Importantly, research reporting a wide variation in laboratory testing behaviour of clinicians for similar syndromes has shown no improvement in clinical outcomes with increasing numbers of tests [17–20].

A variety of studies have been performed with the aim of appropriate use, however most of them have focused on reducing the use of a limited number of laboratory tests [21–24]. The results of a similar organizational use management programme have been published recently and reported that a reduction of 26% in inpatient tests per discharge was managed during a 10-year period [13]. Our results in this study confirmed that hospital committees or organizations may provide a higher impact than focusing on a limited number of specific tests.

Hospital-specific test use review is an important step to define strategies and decide on routes. It has been reported that test use behaviour and practice of medical care and tests might be different according to region and geography [25]. Our initial results showed that creating a hospital committee and performing a test use review provides important information about hospital priorities in appropriate test use and these priorities might differ in different institutions.

Our priority has been identified as prohibition of the clinician test panels because there was a wide variety between panels which can lead to overuse of the tests.

It is important to emphasize that none of the implementations included a prohibition of the laboratory test orders for the clinicians. The clinicians were free to order any of the laboratory tests. However, throughout the course of the study, we welcomed inputs from clinicians caring for patients, about their perceptions regarding the changes in the preordering format. None of them reported any difficulties with the modified system. This indirectly reiterates that many of the tests ordered were perhaps not essential in the first place. Ideally, we would have liked to assess the clinical impact of the modified system in terms of clinical outcomes such as mortality rate, morbidity rates, length of hospitalizations, requirement for enhanced monitoring etc; in order to confirm that patient care was not compromised. However, this was outside the scope of this study. It can be argued that the time of clinicians caring for patients may be increased by having to order lab tests sequentially rather than together. This could theoretically increase costs based on clinician time. However, we have not calculated costs based on this. Informal discussions with physicians suggest that they may not consider this as a limitation to reduce the number of tests ordered initially and proceeding sequentially based on individual patient requirement and test results.

In conclusion, our initial results showed that hospital-based committees including laboratory professionals and clinicians can define hospital based problems and can lead to a standardized approach to test use that can help clinicians considerably reduce laboratory costs through appropriate use of the laboratory tests. In addition, hospital based health technology assessment unit provides a structured ground for such collaborative work.
